# Supplementary material for: The multi-tissue gene expression and physiological responses of water deprived Peromyscus eremicus
Source: BMC Genomics. 2024 Aug 8;25:770. doi: 10.1186/s12864-024-10629-z (PMC11308687; doi:10.1186/s12864-024-10629-z)
Supplement: Supplementary file 2 — Supplementary Material 2 [file 12864_2024_10629_MOESM2_ESM.pdf]

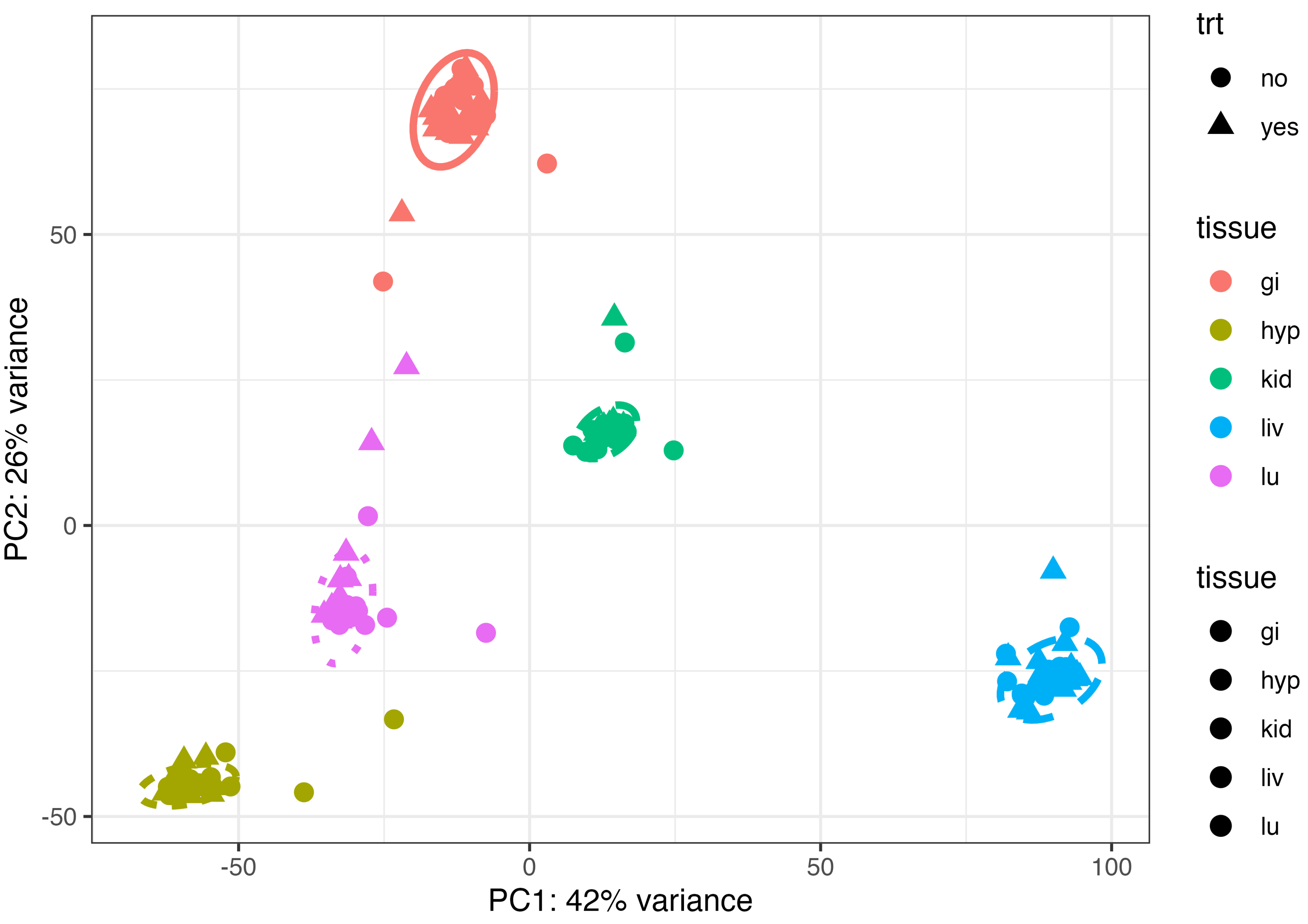

Supplemental File 2 Principal component analysis of gene expression of the lung (lu), liver (liv), gastrointestinal tract (gi), hypothalamus (hyp), and kidney (kid) of Peromyscus eremicus. The axes are labelled with the proportion of the data explained by principal components 1 and 2.
