# Supplementary material for: The multi-tissue gene expression and physiological responses of water deprived Peromyscus eremicus
Source: BMC Genomics. 2024 Aug 8;25:770. doi: 10.1186/s12864-024-10629-z (PMC11308687; doi:10.1186/s12864-024-10629-z)
Supplement: Supplementary file 4 — Supplementary Material 4 [file 12864_2024_10629_MOESM4_ESM.pdf]

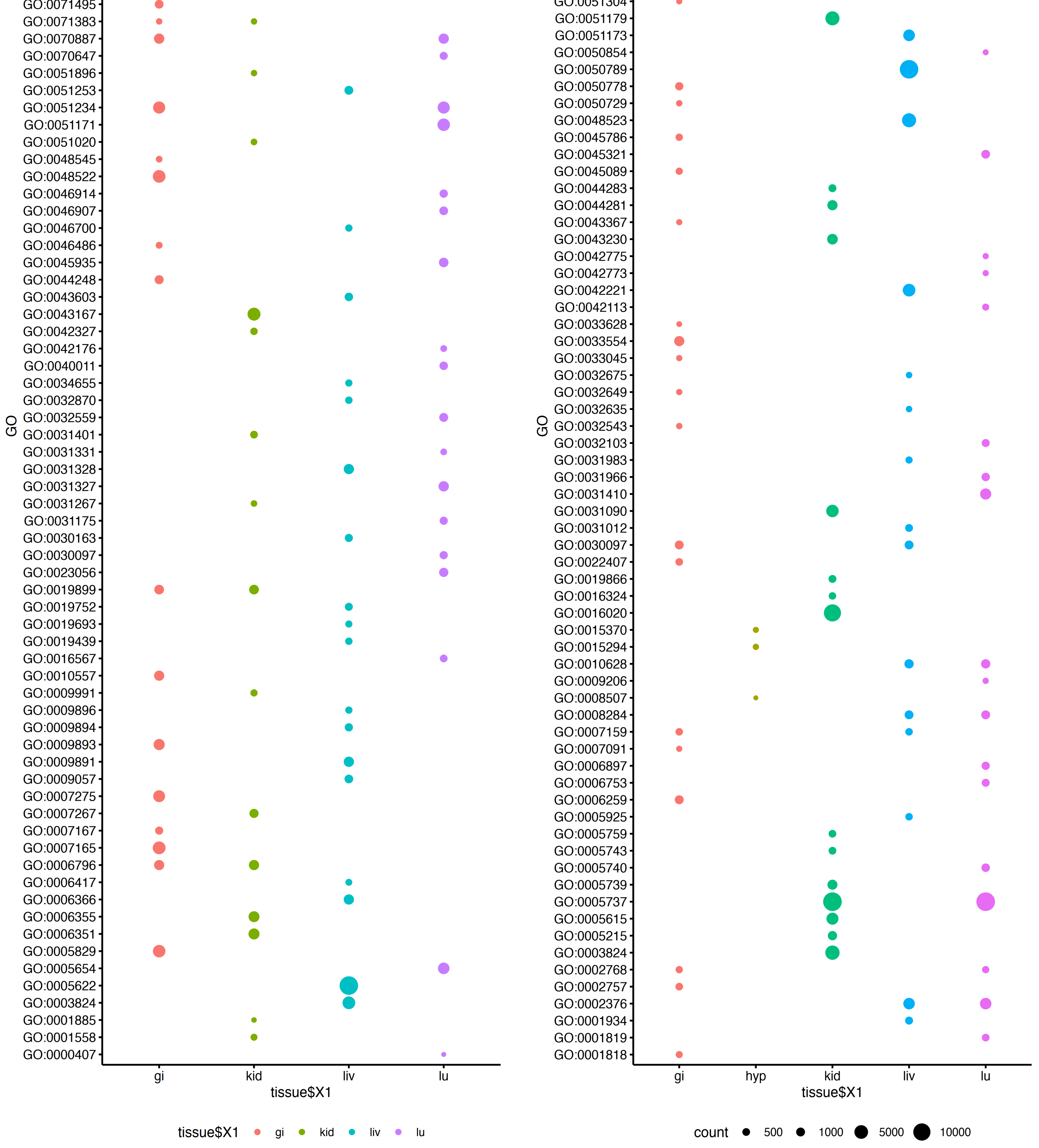

Supplemental File 4 WGCNA results for each gene for each tissue. Each sheet has the module color all gene and what they were assigned to, the table of p values for the Pearson correlation for module color and phenotype, a table of genes and their module assignment for only the significant modules, and a count of genes per significant module. Each sheet is named for the tissue the data came from. CCA WGCNA outlier sheet had the coordinates of the outlier point in the CCA triplot.
